# Contrast of Early Phase Amyloid PET

**DOI:** 10.1002/alz.093758

**Published:** 2025-01-09

**Authors:** Masashi Kameyama, Muneyuki Sakata, Kenji Ishibashi, Tetsuro Tago, Yuto Kamitaka, Jun Toyohara, Kenji Ishii

**Affiliations:** ^1^ Tokyo Metropolitan Institute for Geriatrics and Gerontology, Tokyo Japan

## Abstract

**Background:**

The method of obtaining pseudo‐cerebral blood flow images from the initial minutes of amyloid PET scans has been proposed. This technique proves to be highly valuable as it not only indicates the presence of amyloid accumulation but also reveals sites of neurodegeneration. However, the obtained early images represent the K1 image rather than cerebral blood flow. Consequently, the contrast in early images from amyloid PET tracers is thought to be compromised when compared to cerebral blood flow images.

**Method:**

To enhance the contrast in early images obtained from amyloid PET tracers and thereby improve diagnostic accuracy, we developed a method for contrast recovery and established a methodology for determining the parameter involved in achieving this contrast recovery, drawing insights from a thorough literature review. Additionally, we conducted a comparative analysis involving PiB’s early images and FDG, investigating their respective contributions.

**Result:**

Our study demonstrated a strong correlation between FDG and early images of PiB, with relatively inconspicuous underestimations in high‐blood‐flow regions.

**Conclusion:**

While we theoretically established a method to recover the contrast in early images, our findings suggest that amyloid PET tracers may exhibit a higher permeability‐surface area product than anticipated. This observation raises the possibility that, without correction, these tracers might inherently provide sufficient contrast.

